# Heart rate–corrected systolic ejection time: population-based reference values and differential prognostic utility in acute heart failure

**DOI:** 10.1093/ehjimp/qyad020

**Published:** 2023-09-12

**Authors:** Caroline Morbach, Isabelle Simon, Elisabeth Danner, Götz Gelbrich, Ulrich Stefenelli, Floran Sahiti, Nina Scholz, Vladimir Cejka, Judith Albert, Georg Ertl, Christiane E Angermann, Gülmisal Güder, Stefan Frantz, Peter U Heuschmann, Christoph Maack, Stefan Störk

**Affiliations:** Department Clinical Research and Epidemiology, Comprehensive Heart Failure Center, University Hospital Würzburg, Am Schwarzenberg 15, 97078 Würzburg, Germany; Department Medicine I, University Hospital Würzburg, Oberdürrbacherstr. 6, 97080 Würzburg, Germany; Department Clinical Research and Epidemiology, Comprehensive Heart Failure Center, University Hospital Würzburg, Am Schwarzenberg 15, 97078 Würzburg, Germany; Department Clinical Research and Epidemiology, Comprehensive Heart Failure Center, University Hospital Würzburg, Am Schwarzenberg 15, 97078 Würzburg, Germany; Department Clinical Research and Epidemiology, Comprehensive Heart Failure Center, University Hospital Würzburg, Am Schwarzenberg 15, 97078 Würzburg, Germany; Institute of Clinical Epidemiology and Biometry, University Würzburg, Joseph-Schneider-Str. 2, 97080 Würzburg, Germany; Clinical Trial Center, University Hospital Würzburg, Joseph-Schneider-Str. 2, 97080 Würzburg, Germany; Department Clinical Research and Epidemiology, Comprehensive Heart Failure Center, University Hospital Würzburg, Am Schwarzenberg 15, 97078 Würzburg, Germany; Department Clinical Research and Epidemiology, Comprehensive Heart Failure Center, University Hospital Würzburg, Am Schwarzenberg 15, 97078 Würzburg, Germany; Department Medicine I, University Hospital Würzburg, Oberdürrbacherstr. 6, 97080 Würzburg, Germany; Department Clinical Research and Epidemiology, Comprehensive Heart Failure Center, University Hospital Würzburg, Am Schwarzenberg 15, 97078 Würzburg, Germany; Department Clinical Research and Epidemiology, Comprehensive Heart Failure Center, University Hospital Würzburg, Am Schwarzenberg 15, 97078 Würzburg, Germany; Department Clinical Research and Epidemiology, Comprehensive Heart Failure Center, University Hospital Würzburg, Am Schwarzenberg 15, 97078 Würzburg, Germany; Department Medicine I, University Hospital Würzburg, Oberdürrbacherstr. 6, 97080 Würzburg, Germany; Department Clinical Research and Epidemiology, Comprehensive Heart Failure Center, University Hospital Würzburg, Am Schwarzenberg 15, 97078 Würzburg, Germany; Department Medicine I, University Hospital Würzburg, Oberdürrbacherstr. 6, 97080 Würzburg, Germany; Department Clinical Research and Epidemiology, Comprehensive Heart Failure Center, University Hospital Würzburg, Am Schwarzenberg 15, 97078 Würzburg, Germany; Department Medicine I, University Hospital Würzburg, Oberdürrbacherstr. 6, 97080 Würzburg, Germany; Department Clinical Research and Epidemiology, Comprehensive Heart Failure Center, University Hospital Würzburg, Am Schwarzenberg 15, 97078 Würzburg, Germany; Department Medicine I, University Hospital Würzburg, Oberdürrbacherstr. 6, 97080 Würzburg, Germany; Department Clinical Research and Epidemiology, Comprehensive Heart Failure Center, University Hospital Würzburg, Am Schwarzenberg 15, 97078 Würzburg, Germany; Department Medicine I, University Hospital Würzburg, Oberdürrbacherstr. 6, 97080 Würzburg, Germany; Department Clinical Research and Epidemiology, Comprehensive Heart Failure Center, University Hospital Würzburg, Am Schwarzenberg 15, 97078 Würzburg, Germany; Institute of Clinical Epidemiology and Biometry, University Würzburg, Joseph-Schneider-Str. 2, 97080 Würzburg, Germany; Clinical Trial Center, University Hospital Würzburg, Joseph-Schneider-Str. 2, 97080 Würzburg, Germany; Department Clinical Research and Epidemiology, Comprehensive Heart Failure Center, University Hospital Würzburg, Am Schwarzenberg 15, 97078 Würzburg, Germany; Department Medicine I, University Hospital Würzburg, Oberdürrbacherstr. 6, 97080 Würzburg, Germany; Department Clinical Research and Epidemiology, Comprehensive Heart Failure Center, University Hospital Würzburg, Am Schwarzenberg 15, 97078 Würzburg, Germany; Department Medicine I, University Hospital Würzburg, Oberdürrbacherstr. 6, 97080 Würzburg, Germany

**Keywords:** systolic ejection time, reference values, normal values, risk stratification, prognosis, arterial elastance

## Abstract

**Aims:**

Systolic ejection time (SET) is discussed as a treatment target in patients with heart failure (HF) and a reduced left ventricular (LV) ejection fraction (EF). We derived reference values for SET correcting for its dependence on heart rate (SETc), and explored its prognostic utility in patients admitted with decompensated HF.

**Methods and results:**

SETc was derived in 4836 participants of the population-based STAAB study (mean age 55 ± 12 years, 52% women). There, mean SETc was 328 ± 18 ms, increased with age (+4.7 ms per decade), was shorter in men than women (−14.9 ms), and correlated with arterial elastance (*r* = 0.30; all *P* < 0.001). In 134 patients hospitalized with acute HF, SETc at admission was shorter when compared with the general population and differed between patients with HF with reduced EF (HFrEF; LVEF ≤40%; 269 ± 35 ms), HF with mildly reduced EF (HFmrEF; LVEF 41–49%; 294 ± 27 ms), and HF with preserved EF (HFpEF; LVEF ≥50%; 317 ± 35 ms; *P* < 0.001). In proportional hazard regression, an in-hospital increase in SETc was associated with an age- and sex-adjusted hazard ratio of 0.38 (95% confidence interval 0.18–0.79) in patients with HFrEF, but a hazard ratio of 2.39 (95% confidence interval 1.24–4.64) in patients with HFpEF.

**Conclusion:**

In the general population, SETc increased with age and an elevated afterload. SETc was mildly reduced in patients hospitalized with HFpEF, but markedly reduced in patients with HFrEF. In-hospital prolongation of SETc predicted a favourable outcome in HFrEF, but an adverse outcome in HFpEF. Our results support the concept of a U-shaped relationship between cardiac systolic function and risk, providing a rationale for a more individualized treatment approach in patients with HF.

## Graphical Abstract

**Graphical Abstract:**
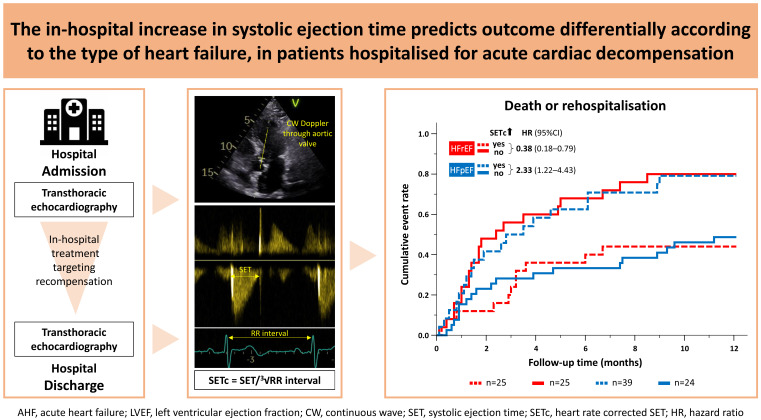

## Introduction

Heart failure (HF) may be categorized by left ventricular (LV) ejection fraction (EF) into HF with reduced (HFrEF), mildly reduced (HFmrEF), or preserved EF (HFpEF).^1^ While patients with HFrEF benefit from drugs interfering with neuroendocrine activation, this is not the case in patients with HFpEF.^1^ Once hospitalized, in-hospital and 1-year mortality are substantially elevated across the entire EF spectrum,^2^ in particular when repeated hospitalizations or early rehospitalization after discharge occur,^3^ emphasising the need to optimise haemodynamics of HF patients during recompensation.^1^

The treatment of patients with acute HF (AHF) remains challenging, since most approaches to improve in-hospital outcomes failed.^4^ Myosin activators, such as omecamtiv mecarbil^5^ and danicamtiv,^6^ prolong myocyte shortening without affecting intracellular Ca^2+^ concentrations,^7^ thereby extending LV systolic ejection time (SET) and increasing stroke volume (SV) and cardiac output.^5,8^ In patients with chronic HFrEF, omecamtiv reversed cardiac remodelling, reduced *N*-terminal pro B-type natriuretic peptide (NT-proBNP), and improved cardiovascular mortality and HF-related hospitalization risk in the Phase 3 Global Approach to Lowering Adverse Cardiac Outcomes through Improving Contractility in Heart Failure (GALACTIC-HF) trial.^9^ These benefits were most pronounced in patients with lower LVEF or hospitalized patients with elevated NT-proBNP levels.^5,9^ At the other end of the HF (and LVEF) spectrum, the myosin ATPase *inhibitor* mavacamten *reduces* LVEF and improves symptoms, exercise capacity, and LV geometry in patients with hypertrophic obstructive cardiomyopathy,^10^ a hereditary form of HFpEF characterized by hypercontractility.

As myosin activators improve SV through prolonging SET,^5^ this parameter received increasing attention as an easily accessible imaging biomarker in HF.^11^ SET is reduced in patients with HFrEF and correlates with decreased LVEF, SV, and global longitudinal strain.^11^ Shortened SET was an independent predictor of incident HF in a community-based cohort of individuals without prior HF and predicted death or hospitalization for HF in patients with HFrEF, but not HFpEF.^11^

While these studies were performed in patients with stable HF, it is unknown how SET is altered during acute cardiac decompensation, and whether recompensation modulates SET in conjunction with other haemodynamic parameters. Since SET strongly depends on heart rate (HR),^11^ previous studies either accounted for HR through adjustment or applied a SET index, which has been derived from small cohorts of healthy individuals and patients with cardiac diseases in the early 1960s using electrocardiogram, phonocardiogram, and carotid arterial pulse tracing.^11^ However, up to now, valid echocardiography-based reference values corrected for HR are lacking.^11^

Here, using state-of-the-art echocardiography, we aimed to (i) provide HR-corrected reference values for SET from a population-based cohort and assess determinants of SET in individuals without HF, (ii) determine SET in AHF patients across the entire LVEF spectrum and assess the association of SET with parameters of systolic and diastolic function, and (iii) determine the differential prognostic utility of dynamic SET modification during hospitalization.

## Methods

### Healthy controls

The population-based *Characteristics and Course of Heart Failure Stages A-B and Determinants of Progression* (STAAB) Cohort Study recruited individuals without self-reported HF from the general population of Würzburg, Germany, aged 30–79 years and stratified for age and sex. The detailed study design and methodology have been published.^12^ All study related procedures were subjected to a rigid and regular quality control process.^12^ All participants underwent an extensive, pre-specified transthoracic echocardiography protocol (Vivid S6 or Vivid E95; GE Healthcare, Horten, Norway) performed by dedicated certified personnel that was quality controlled on a regular basis.^13^ A subset of individuals, who were free from cardiovascular risk factors including hypertension, smoking, obesity, dyslipidaemia, diabetes mellitus, and known cardiovascular disease, were defined as ‘apparently healthy’ (for definitions refer to Supplementary methods). This subgroup was selected to derive reference values.

### AHF patients

As part of a larger research initiative, the *Comprehensive Heart Failure Center* (CHFC) identifies and phenotypes consecutive patients admitted to the University Hospital Würzburg for AHF. Selection criterion is the presence of AHF (first or recurrent event) diagnosed by the respective physician in-charge based on signs, symptoms, and the results of clinically indicated diagnostic tests according to current guidelines.^1,14^ Exclusion criteria are high output HF, cardiogenic shock, or being listed for high-urgency heart transplant. Patients were treated according to current guidelines (best clinical practice^1,14^), and discharged based on their treating physician’s decision. Survival status as well as information regarding potential rehospitalization was obtained after 6 months during an outpatient visit, by telephone interview, or based on information from general practitioners, relatives, or registration authorities. In the present analysis, we included patients providing pairs of echocardiograms recorded within 3 days from admission and prior to discharge. We used the stored echocardiograms (Vivid E9 or Vivid E95; GE Healthcare) performed in the clinical context.

Both studies comply with the Declaration of Helsinki and received positive votes from the Ethics Committee of the Medical Faculty as well as from the data protection officer of the University of Würzburg (votes #98/13 and #55/14).

### Determination of SET, HR, peripheral elastance, and echocardiography parameters

The flow through the aortic valve was assessed using a continuous-wave Doppler from an apical five- or three-chamber view (as individually appropriate), and one still-frame with up to five R-R intervals was stored. We measured SET from the start to the end of the transaortic flow, hence from opening to closure of the aortic valve, and determined the R-R interval to calculate the respective HR for each SET measurement. Up to five SET and R-R intervals were measured and the mean of the respective measurements entered the statistical analyses. To assess interobserver variability, 40 random echo scans were measured from independent observers (I.S. and E.D.), blinded to the other observer’s results. LV end-diastolic (LVEDV) and end-systolic volumes (LVESV) were measured in apical four- and two-chamber views by Simpson’s method, and SV (SV = LVEDV − LVESV), LVEF (=SV/LVEDV) and cardiac output (SV × HR) were calculated. In cases of suboptimal image quality, LVEF was estimated visually. Early mitral inflow velocity (*E*) was assessed using pulsed-wave (PW) Doppler with the acquisition window positioned at the mitral valve leaflet tips, early diastolic velocity of the basal septal and lateral mitral annulus (*e*´) was assessed using PW and tissue Doppler and averaged for further analyses. Using an apical three-, four-, and two-chamber views, we assessed LV longitudinal strain. Finally, arterial elastance (Ea = 0.9×SBP/SV) and end-systolic elastance (Ees = 0.9×SBP/LVESV) were calculated as described previously.^15^

### Data analysis

Statistical analysis was performed using SPSS (Version 26; SPSS Inc., Chicago, IL, USA and R, Version 4.3). Values were summarized as frequencies (per cent), mean (standard deviation), and median (quartiles), as appropriate. Observer variability was assessed in 40 STAAB participants using Bland–Altman 95% limits of agreement (LoA). To obtain HR-corrected SET (SETc), we tried several approaches in analogy to transformations used for electrocardiographic QT interval correction.^16^ Locally weighted scatterplot smoothing (LOWESS) was used to inspect deviations.

Pairs of measurements were compared using Wilcoxon’s test. Reference values were derived from apparently healthy individuals. The prognostic value was assessed by uni- and multivariable Cox proportional hazards regression, and hazards with 95% confidence intervals (CI) were reported. Results are considered exploratory; hence, no adjustment for multiple testing was introduced. All tests were performed two-sided, and *P*-values <0.05 were considered statistically significant.

## Results

### SET in the general population

We analysed 4965 individuals included in the STAAB study.^12,17^ SET could be determined in 4836 participants (feasibility: 97%). Their mean age was 55 ± 12 years, 52% were women, mean body mass index was 26.6 ± 5.2 kg/m², mean LVEF was 60 ± 5%, and 99% were in sinus rhythm. The average HR was 68 ± 11 min^−1^, and the mean SET was 318 ± 26 ms. Reproducibility of SET measurement was excellent (95% LoA: −18.5, 14.3 ms). SET was strongly and inversely correlated with HR (*r* = −0.59, *P* < 0.001). The best model fit for SETc was apparent using a Fridericia-like approach, i.e. SET/^3^√R-R interval. Using this equation, the linear regression line almost converged to the LOWESS regression line (see Supplementary data online, *Figure e1*).

**Figure 1:**
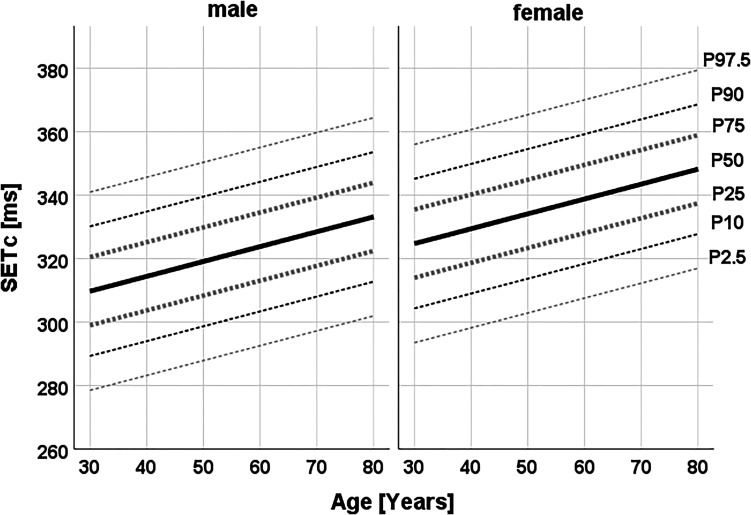
Sex-specific percentiles of SETc according to age, derived from a population-based cohort of individuals free from heart failure (*n* = 966 *apparently healthy* individuals; 49 ± 11 years, 61% women).

SETc equalled 329 ± 22 ms in the total population. Both SET and SETc increased with age (+5.1 and +4.7 ms per decade, respectively; both *P* for trend <0.001). Furthermore, compared with women, men had shorter SET (−6.6 ms) and SETc (−14.9 ms; both *P* < 0.001). Age- and sex-specific reference values for SET and SETc were generated from the subgroup of 966 apparently healthy individuals (49 ± 11 years, 61% women). The detailed values per age and sex subgroup are shown in *Figure 1* and Supplementary data online, *Tables e1* and *e2*.

Since pre-clinical studies had suggested that an acute increase in cardiac afterload may potentiate cardiac contractility through the so-called Anrep effect^18^ and thereby prolongs SET,^19^ we determined arterial (Ea) and end-systolic elastance (Ees).^15^ The former informs on afterload, and the latter is an indicator of LV systolic performance independent of arterial pre- and afterload. Both elastance variables increased with age: *r* = 0.30 for Ea, *r* = 0.20 for Ees; both *P* < 0.001. In both men and women, SETc correlated positively with Ea (total population, *r* = 0.19; *P* < 0.001) and Ees (*r* = 0.21; *P* < 0.001), respectively.

### SETc in patients with AHF

Between September 2014 and January 2017, 623 consecutive patients with AHF (75 ± 11 years, 40% women) entered the study. Their main clinical characteristics are summarized in *Table 1* and Supplementary data online, *Table e3*. Serial echocardiograms to determine SET were available in 134 patients (73 ± 11 years, 37% women; *Table 1*) and had been collected at 1 (0, 2) day(s) after admission and 1 (0, 3) day(s) prior to discharge. These *n* = 134 patients entered further analyses. Of those, 52 patients had an LVEF ≤40% at discharge (i.e. HFrEF), 16 patients had an LVEF of 41–49% (i.e. HFmrEF), and 66 patients had an LVEF of ≥50% (i.e. HFpEF). Patients with HFrEF vs. HFmrEF and HFpEF were younger, but had a similar sex distribution. HFrEF patients had higher HR and NT-proBNP levels, larger LVEDV and lower SV when compared with HFmrEF and HFpEF patients, respectively. Cardiac output was equally low in all three groups (*Table 2*).

**Table 1:** Baseline characteristics of patients admitted to the hospital with AHF

	Patients with HFrEF *n* = 52	Patients with HFmrEF *n* = 16	Patients with HFpEF *n* = 66	*P*-value across types of HF
Female sex	16 (31)	5 (31)	28 (42)	0.382
Age (years)	67 (14)	77 (6)	76 (9)	0.001^ab^
Body mass index (kg/m^2^)	29.1 (13.5)	30.3 (6.1)	30.1 (7.6)	0.670
De novo HF	7 (13)	1 (6)	13 (20)	0.354
Coronary disease	22 (43)	9 (56)	26 (41)	0.528
Diabetes mellitus	33 (66)	11 (69)	36 (55)	0.408
Atrial fibrillation	24 (48)	10 (63)	29 (45)	0.439
NYHA functional class				0.459
I/II	4 (8)	0 (0)	3 (5)	
III/IV	48 (92)	16 (100)	62 (95)	
Pharmacotherapy for HF (at admission)				
Beta-blocker	38 (73)	12 (75)	43 (65)	0.569
ACEi/ARB	26 (50)	8 (50)	36 (55)	0.871
Sacubitril/valsartan	2 (4)	0 (0)	0 (0)	0.202
MRA	19 (37)	5 (31)	10 (15)	0.025
Diuretics	46 (88)	15 (94)	63 (95)	0.350
NT-proBNP (pg/mL)	6152 (4124, 10428)	3982 (2457, 5808)	2968 (1249, 6397)	0.001^b^
eGFR (mL/min/1.73 m^2^)	52 (39, 66)	59 (40, 69)	47 (33, 68)	0.575
Glucose (mg/dL)	131 (109, 168)	116 (110, 136)	120 (102, 171)	0.640
Total cholesterol (mg/dL)	133 (113, 152)	151 (98, 167)	145 (132, 165)	0.589
Serum sodium (mmol/L)	138.9 (4.0)	138.1 (5.0)	139.4 (4.4)	0.496
Serum potassium (mmol/L)	4.4 (0.5)	4.4 (0.7)	4.4 (0.5)	0.698
LVEF (%)	24.8 (7.8)	47.4 (6.6)	59.6 (10.0)	-

Values indicate *n* (%), mean (SD), or median (Q1, Q3), as appropriate. Pairwise comparisons, *P* < 0.05: a = HFrEF vs. HFmrEF, b = HFrEF vs. HFpEF, c = HFmrEF vs. HFpEF.

ACEi, angiotensin conversion enzyme inhibitor; ARB, angiotensin receptor blocker; HFrEF, HF with reduced EF (LVEF ≤ 40%); HFmrEF, HF with mildly reduced EF (LVEF 41–49%); HFpEF, HF with preserved EF (LVEF ≥50%); MRA, mineralocorticoidreceptor antagonist; NYHA, New York Heart Association functional class; NT-proBNP, *N*-terminal pro B-type natriuretic peptide; eGFR, estimated glomerular filtration rate; HbA1c, glycosylated haemoglobin c; LVEF, left ventricular ejection fraction.

**Table 2:** Change in clinical and echocardiographic parameters from admission to discharge in patients admitted to the hospital for AHF according to LVEF at discharge

	Admission				Change from admission to discharge, Δ [95% CI]			
	HFrEF *n* = 52	HFmrEF *n* = 16	HFpEF *n* = 66	*P*-value	HFrEF *n* = 52	HFmrEF *n* = 16	HFpEF *n* = 66	*P*-value
Body weight (kg)	87 (20)	90 (21)	84 (22)	0.363	−4.4 (−5.7, −2.8)	−3.7 (−6.2, −0.9)	−2.6 (−3.8, −1.9)	0.032^b^
NT-proBNP (pg/mL)	6152 (4124, 10428)	3982 (2457, 5808)	2968 (1249, 6397)	0.001^b^	−3942 (−5737, −1922)	−2008 (−3800, 423)	−2392 (−3490, −1063)	0.061^b^
HR (min^−1^)	84 (19)	75 (24)	74 (17)	0.016^b^	−12 (−17, −7)	−2 (−11, 8)	−7 (−11, −3)	0.059
SET (ms)	244 (39)	279 (36)	300 (46)	<0.001^ab^	20 (11, 30)	−5 (−21, 10)	10 (−0, 19)	0.025^a^
SETc (ms)	269 (35)	294 (27)	317 (35)	<0.001^abc^	9 (1, 17)	−5 (−21, 10)	1 (−7, 9)	0.110
LVEF (%)	25 (8)	46 (6)	60 (11)	<0.001^abc^	11 (7, 15)	3 (−1, 8)	1 (−2, 3)	0.004^b^
LVEDV (mL)	161 (120, 208)	100 (64, 134)	79 (67, 114)	<0.001 ^ab^	−9 (−20, 3)	−27 (−55, 1)	−5 (−12, 4)	0.409
SV (mL)	39 (27, 48)	46 (30, 69)	50 (38, 72)	0.002^b^	4 (+0, 8)	−14 (−27, −1)	−1 (−6, 3)	0.065^a^
Cardiac output (L/min)	3.2 (2.2, 4.2)	3.7 (2.2, 4.5)	3.6 (2.8, 4.8)	0.222	−0.2 (−0.6, 0.2)	−1.1 (−2.0, −0.3)	−0.5 (−0.9, −0.1)	0.334
*E*/*e*´	19 (13, 25)	20 (16, 20)	17 (12, 22)	0.590	4 (−7,12)	13 (+0, 25)	2 (−1, 5)	0.577
TRmaxPG (mmHg)	41 (11)	41 (15)	44 (14)	0.502	−3 (−6, + 0)	−4 (−10, 2)	−6 (−9, −3)	0.539
Ea (mmHg/mL)	2.85 (2.20, 3.76)	1.94 (1.72, 3.50)	2.43 (1.67, 3.07)	0.038^b^	−0.46 (−0.82, −0.05)	0.63 (−0.46, 1.64)	0.06 (−0.36, 0.43)	0.114
Ees (mmHg/mL)	0.93 (0.65, 1.20)	2.28 (1.43, 2.95)	3.87 (2.76, 5.22)	<0.001^abc^	0.07 (−0.06, 0.18)	0.45 (−0.75, 1.60)	0.18 (−0.40, 0.71)	0.706

Value are given as mean (SD) or median (Q1, Q3), as appropriate. The difference (Δ) admission to discharge was calculated subtracting the admission value from the discharge value; hence, a difference >0 indicates a prolongation of SET or SETc from admission to discharge, respectively. The respective information was available in ≥80% of participants, except for: ΔEa (*n* = 94), ΔLVEDV (*n* = 96), and *E*/*e*´ (*n* = 66). Pairwise comparisons, *P* < 0.05: a = HFrEF vs. HFmrEF, b = HFrEF vs. HFpEF, c = HFmrEF vs. HFpEF.

HFrEF, HF with reduced EF (LVEF ≤ 40%); HFmrEF, HF with mildly reduced EF (LVEF41–49%); HFpEF, HF with preserved EF (LVEF ≥ 50%); HR, heart rate; NT-proBNP, *N*-terminal pro B-type natriuretic peptide; SET, systolic ejection time; SETc, HR-corrected SET; LVEF, left ventricular ejection fraction; LVEDV, left ventricular end-diastolic volume; SV, stroke volume; *E*, early mitral inflow velocity; *e*´, early diastolic velocity of the mitral annulus; TRmaxPG, maximal tricuspid regurgitation pressure gradient; Ea, afterload; Ees, left ventricular end-systolic elastance/contractility.

**Figure 2:**
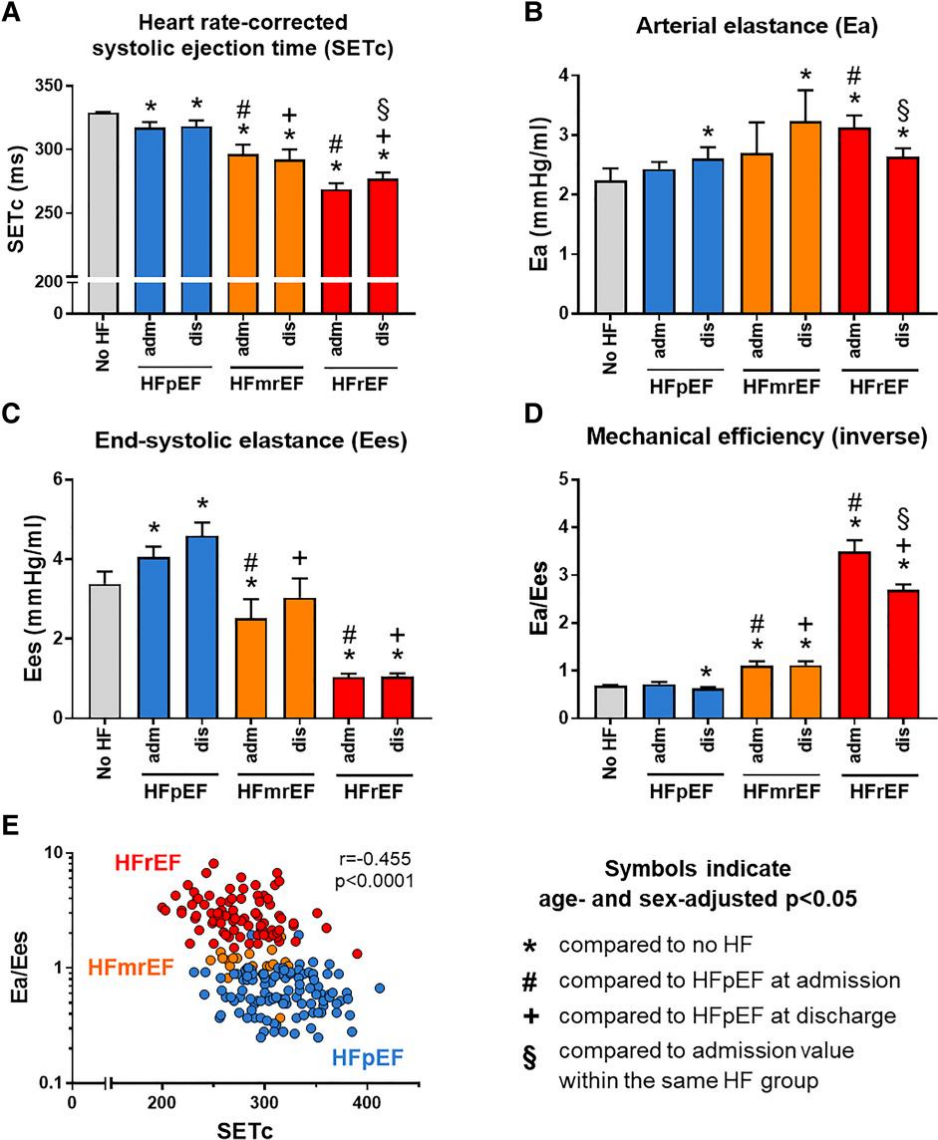
(*A*) The HR-corrected systolic ejection time (SETc), (*B*) arterial elastance, (*C*) left ventricular end-systolic elastance, and (*D*) mechanical efficiency as well as (E) the correlation of mechanical efficiency and SETc in individuals without HF (STAAB cohort) and in patients admitted with AHF displayed by HF subgroup. STAAB, population-based *Characteristics and Course of Heart Failure Stages A-B and Determinants of Progression* Cohort Study; SETc, HR corrected systolic ejection time; Ea, arterial elastance; Ees, left ventricular end-systolic elastance; Ea/Ees, mechanical efficiency; no HF, individuals participating in the STAAB study (*n* = 4836); HFrEF, HF with reduced EF (LVEF ≤ 40%, *n* = 52); HFmrEF, HF with mildly reduced EF (LVEF 41–49%, *n* = 16); HFpEF, HF with preserved EF (LVEF ≥50%, *n* = 66).

SETc at admission differed significantly between HF subgroups: patients with HFpEF had the longest SETc (317 ± 35 ms) when compared with HFmrEF (294 ± 27 ms) and HFrEF (269 ± 35 ms, *P* < 0.001), respectively. Even though, SETc in HFpEF patients was of significantly shorter duration when compared with SETc in individuals without HF (STAAB cohort), with a mean age- and sex-adjusted difference of 20 ms (95% CI 11–29 ms, *P* < 0.001; *Figure 2A*).

Upon admission, Ea, a marker of afterload, was higher in patients with HFrEF when compared with HFpEF as well as when compared with individuals without HF (*Figure 2B*). In contrast, Ees, a marker of systolic cardiac performance, was lower in HFrEF and HFmrEF when compared with HFpEF (*Figure 2C*). When compared with individuals without HF, Ees was higher in HFpEF but lower in HFmrEF and HFrEF patients, respectively (*Figure 2C*). The ratio of Ea/Ees was significantly higher in HFrEF and HFmrEF when compared with HFpEF as well as when compared with individuals without HF, respectively (*Figure 2D* and *Table 2*), indicating lower mechanical efficacy in patients with HFmrEF and HFrEF.

### Dynamic alterations of haemodynamics during recompensation

Between admission and discharge, all three patient groups showed a significant weight loss (−4.4 vs. −3.7 and −2.6 kg), which was significantly higher in patients with HFrEF when compared with HFpEF (*P* = 0.032; *Table 2*). This was paralleled by a significant decrease in HR in patients with HFrEF and HFpEF. In HFrEF, we further observed a significant increase in LVEF and SV as well as a decrease in Ea and Ea/Ees, whereas these alterations were not evident in HFpEF (*Table 2*). With decongestive efforts during the in-hospital period, we observed a significant increase in SETc in patients with HFrEF but not with HFmrEF or HFpEF (*Table 2* and *Figure 2*). In patients with HFrEF, the change in SETc correlated with the change in NT-proBNP (*r* = −0.43, *P* = 0.005), while there was no such correlation in HFpEF patients (*r* = −0.03, *P* = 0.834).

### Prognostic utility of SETc

**Figure 3:**
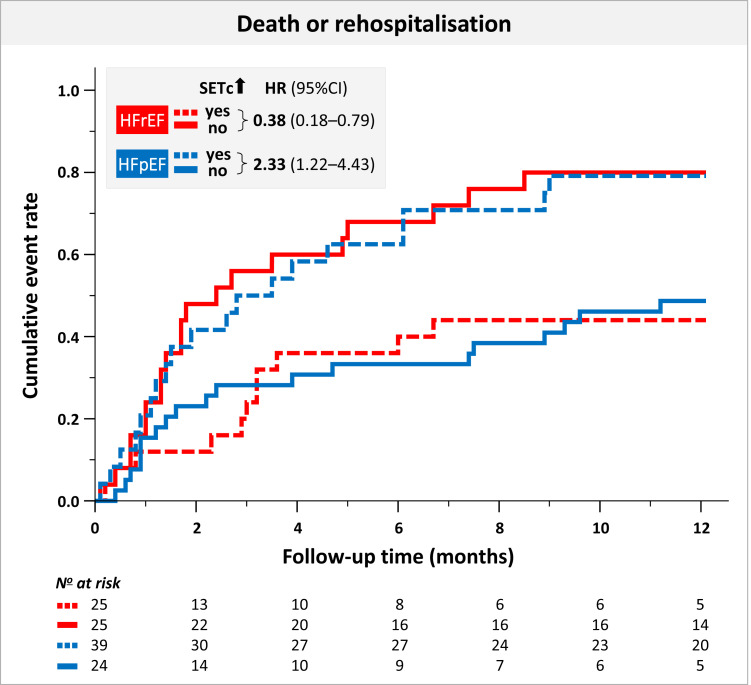
Cumulative event rate in patients hospitalized with AHF with and without an in-hospital increase in SETc according to HF subgroup. SETc, HR corrected systolic ejection time, HR, hazard ratio, CI, confidence interval, HFrEF, HF with reduced EF (LVEF ≤ 40%); HFpEF, HF with preserved EF (LVEF ≥50%).

In the 12 months after discharge, 67 patients experienced the primary endpoint. While in patients with HFrEF, SETc at discharge *per se* did not predict the risk of the primary endpoint of 12-month death or hospitalization for HF, the dynamic prolongation of SETc from admission to discharge was associated with a favourable prognosis (hazard ratio 0.87, 95% CI 0.78–0.96, *P* = 0.006; *Table 3*). In contrast, in patients with HFpEF, longer SETc at discharge was associated with an increased risk for death or rehospitalization (hazard ratio 1.15, 95% CI 1.05–1.25, *P* = 0.003). Furthermore, an increase in SETc in patients with HFpEF was associated with a trend towards a higher risk (hazard ratio 1.01, 95% CI 0.99–1.18, *P* = 0.067; *Table 3*). Accordingly, compared with patients with no increase in SETc during hospitalization, an increase in SETc between admission and discharge was associated with a reduction of the risk by 62% in patients with HFrEF, but a 2.39-fold increase in patients with HFpEF (*Figure 3*).

**Table 3:** Prognostic value of SETc regarding 12-month prognosis in patients hospitalized with AHF

	Death or rehospitalization	
	Hazard ratio per 10 ms increment in SETc	*P*-value
Admission		
HFrEF	1.09 (1.00, 1.20)	0.052
HFmrEF	0.92 (0.67, 1.25)	0.573
HFpEF	1.05 (0.95, 1.15)	0.336
Discharge		
HFrEF	1.00 (0.89, 1.11)	0.927
HFmrEF	1.26 (0.94, 1.70)	0.122
HFpEF	1.15 (1.05, 1.25)	0.003
Difference (Δ) admission to discharge		
HFrEF	0.87 (0.78, 0.96)	0.006
HFmrEF	1.24 (0.96, 1.61)	0.105
HFpEF	1.01 (0.99, 1.18)	0.067

Hazard ratios were calculated using Cox regression and adjusting for age and sex. The difference (Δ) admission to discharge was calculated subtracting the admission value from the discharge value; hence, a difference >0 indicates a prolongation of SET or SETc from admission to discharge, respectively.

## Discussion

Our study revealed that, in patients with AHF, the simple iterative assessment of SETc during hospitalization provides important information that may aid tailoring care in patients with AHF. In a first step, we derived HR corrected reference values for SET from a population-based cohort. In this cohort, longer SETc was associated with increasing age and a higher afterload. This is compatible with the concept that SETc increases with advancing age in relation to an increased afterload. When determining SETc in a sample of patients with AHF, we observed that (i) SETc was reduced in patients with HFpEF compared with individuals of the general population, (ii) SETc was further shortened in patients with HFmrEF and HFrEF, (iii) SETc increased during in-hospital recompensation in patients with HFrEF, but not in HFmrEF or HFpEF, and (iv) prolongation of SETc in patients with HFrEF was associated with a lower risk of rehospitalization and death, while SETc prolongation in patients with HFpEF predicted an adverse outcome.

### SET in the normal population

Due to technological developments, the methods to measure SET changed substantially over the last decades.^11^ First studies in the 1960s measured SET by electrocardiogram, central arterial pulse, and phonocardiogram. In the 1980s, phonocardiogram was replaced by M-mode echocardiography, followed more recently by pulsed-wave or colour tissue Doppler imaging.^11^ Here, we derived SET with high feasibility and low interobserver variability from continuous-wave Doppler tracings of transaortic flow, implying that SET can be reliably determined both in clinical routine and trials. To correct for HR, authors previously calculated the LV ejection time index by adding a multiple of HR to SET.^11^ However, since this method is inaccurate and never entered clinical routine, we applied the cubic root Fridericia’s formula,^16^ because it is well established for HR correction of the QT interval. Based on a large population-based sample of individuals without HF, we were able to compute SETc reference values for subjects aged 30–79 years. We observed that SETc was ∼15 ms longer in women than in men, and steadily increased with age by ∼5 ms per decade, which both is in agreement with reports on uncorrected SET.^11^

To better understand the age-dependent increase in SETc, we determined afterload by arterial elastance (Ea) in the same population. In fact, Ea increased with age, and this increase correlated with the increase in SETc. The heart is able to intrinsically adjust contractility to alterations in haemodynamic load. While the Frank–Starling mechanism is a sarcomere-based mechanism to increase cardiac output in response to elevated *preload*,^20^ the so-called Anrep effect^18^ describes the adaptation of contractility to increased *afterload* through sarcomeric phosphorylation by Ca^2+^/calmodulin-dependent protein kinase II (CaMKII), likely secondary to oxidative stress.^19^ In fact, elevations in cardiac *afterload* provoke mitochondrial reactive oxygen species emission in cardiac myocytes,^21^ which then may activate CaMKII.^22^ While CaMKII-dependent phosphorylation of Ca^2+^ handling proteins increases inotropy in the short term, it causes cardiac hypertrophy and, potentially, cell death in the longer term.^8^ While the present clinical results cannot clarify the underlying mechanisms, these experimental data may yet provide a plausible explanation, how an increase in cardiac afterload prolongs SET in aged individuals who are still unaffected by HF. At the same time, prolonged SETc may serve as an indicator of an increased cardiac risk, since CaMKII activation is a known prohypertrophic factor that may contribute to the development of HF.^23^ We consider the Anrep effect^19^ a more likely explanation of age-dependent SETc prolongation than an increase in sympathetic tone, since adrenergic stimulation *shortens* SET.^24^

### SET in congested and decongested HF

At admission, all three HF groups showed equally reduced cardiac output, elevated LV filling pressures and increased pulmonary artery pressure, but we found differential haemodynamic situations: HFrEF patients had significantly larger LV volumes, lower SVs, and reduced Ees (i.e. a marker of systolic cardiac performance) as well as higher HR and higher Ea/Ees (i.e. a surrogate which inversely correlates to mechanical efficiency), when compared with HFpEF. HFmrEF patients ranged between these HF phenotypes.

With recompensation efforts, we observed an in-hospital decrease in weight and in NT-proBNP in all three HF groups, which was most pronounced in HFrEF. Furthermore, cardiac recompensation efforts during hospitalization prolonged SETc, reduced afterload, and improved mechanical efficiency in patients with HFrEF, but neither with HFmrEF nor HFpEF. Since the prolongation of SETc, which indicates improved systolic function in conditions of forwards failure, was associated with improved outcomes after discharge of patients with HFrEF, these data emphasize that the haemodynamic optimization, which allows improvement of contraction during hospitalization (reported by SETc) is key for long-term outcome, and interventions that reduce afterload (such as sacubitril/valsartan^25^) may exert their beneficial effects by relieving cardiac afterload and thus allowing SETc to prolong.

In contrast, the fact, that the prolongation of SETc was associated with adverse outcome in patients with HFpEF, may be related to shortening of diastolic filling time and thereby aggravated haemodynamic compromise in this patient population. Recently, a U-shaped association between LVEF and mortality risk was reported in patients with HF, identifying a phenotype of ‘supranormal LVEF’ to impose increased cardiac risk.^26^ Similarly, Haiden *et al*.^27^ observed a U-shaped relationship of SET with 8-year survival in 852 patients with suspected coronary artery disease. In patients with HFpEF, machine-learning-based analyses of right heart catheterization and echocardiography data revealed distinct phenotypes within the spectrum of HFpEF, with one rather HFrEF-like phenotype and one with *supranormal* LV function, which was associated with elevated systemic and pulmonary artery resistance.^28^ A prototype of supranormal LV function is hypertrophic cardiomyopathy, where mutations of genes encoding sarcomeric proteins may cause supranormal systolic and compromised diastolic function at increased energetic cost.^29^ In these patients, *reducing* inotropy with mavacamten improved symptoms, increased functional capacity, lowered NT-proBNP levels, and reversed maladaptive cardiac remodelling despite—or rather due to—lowering LVEF.^10^ Together, these data indicate that across the heterogeneous spectrum of acute and chronic HF phenotypes, both hypo- and hyperdynamic phenotypes exist, which may require specific diagnostic and therapeutic approaches.

### Strengths and limitations

The current study provides hypothesis-generating mechanistic insights into the alterations accompanying cardiac deterioration leading to AHF and the impact of subsequent recompensation, in relation to HF subgroups based on serial echocardiograms. The timely acquisition of high-quality echocardiograms in AHF patients was challenging and was feasible only in a subgroup of the cohort. In particular, the sample of patients with HFmrEF was rather small. Since the underlying pathophysiology in HFmrEF varies, we abstained from mixing them with either the HFrEF or HFpEF group, but chose to exclude them in order to not dilute the results.

A major strength of the current manuscript is the combination of data from patients with AHF and a large, well-characterized and representative population-based sample. Thereby, we were able to provide reference values for SET and SETc. The STAAB cohort and the AHF patient sample have been recruited from the same catchment area allowing for direct comparisons.

### Potential therapeutic implications

Patients with AHF have a high risk of death not only during hospitalization, where outcome depends strongly on haemodynamic variables, but also in the year following hospitalization.^2^ Therefore, optimal cardiac recompensation is key to improve not only in-hospital outcome, but also long-term prognosis in these patients. While several drugs designed to specifically treat patients with AHF failed to improve long-term outcome,^4^ more recent trials revealed that in-hospital initiation of compounds recommended for patients with *chronic* HFrEF, such as sacubitril/valsartan^25^ or sodium-glucose cotransporter 2 inhibitors,^30^ improved 90-day outcomes of patients with AHF. While these compounds provided benefit *independent* of LVEF and other haemodynamic parameters amongst patients with HFrEF, the myosin activator omecamtiv mecarbil, which prolongs SET, was more effective in patients with lower LVEF, lower blood pressure, and those patients hospitalized with high NT-proBNP levels.^9,31^ These data suggest that a more personalized approach towards AHF that takes into account haemodynamic variables during cardiac decompensation and recompensation is required to optimise medical treatment for this patient population at particular risk. Our results might thus contribute to a better understanding of the pathophysiology of AHF and recompensation, respectively, and support the development of more individualized treatment approaches.

## Conclusion

We derived SETc from a population-based cohort. In healthy individuals, the time interval for SETc increased with age and with higher vascular afterload. The serial assessment of the novel echocardiography-derived parameter SETc, monitoring its dynamic regulation in AHF patients, predicted outcome, and may thus serve to identify patients who benefit best from interventions prolonging SET. Prolongation of SETc during cardiac recompensation is associated with beneficial outcomes in patients with HFrEF, but adverse outcomes in patients with HFpEF, supporting the concept of a U-shaped relationship between cardiac systolic function and risk, paving the way towards more individualized treatment approaches of patients with acute and chronic HF.

## Supplementary Material

qyad020_Supplementary_Data

## Data Availability

Data can be made available upon reasonable request.

## Lead author biography

Caroline Morbach currently serves as cardiologist and echocardiography specialist at the University Hospital Würzburg, Germany. She founded the Academic Core Lab Ultrasound-based Cardiovascular Imaging at the Comprehensive Heart Failure Center Würzburg and established internal training and certification algorithms to ensure high-quality standards in clinical and scientific echocardiography. She is a fellow of the European Association of Cardiovascular Imaging (EACVI) and certified specialist in transthoracic and transoesophageal echocardiography both, from the EACVI as well as from the German ultrasound association (DEGUM). She further has long-term experience in clinical research and care for patients with advanced heart failure and patients with cardiac amyloidosis.
