# Supplementary material for: Heart rate–corrected systolic ejection time: population-based reference values and differential prognostic utility in acute heart failure
Source: Eur Heart J Imaging Methods Pract. 2023 Sep 12;1(2):qyad020. doi: 10.1093/ehjimp/qyad020 (PMC11195710; doi:10.1093/ehjimp/qyad020)
Supplement: qyad020_Supplementary_Data [file qyad020_Supplementary_Data.docx]

**Supplementary material to**

**Heart rate corrected systolic ejection time: population-based reference values and differential prognostic utility in acute heart failure**

Caroline Morbach MD^1,2^, Isabelle Simon^1^, Elisabeth Danner^1^, Götz Gelbrich PhD PhD^1,3,4^, Ulrich Stefenelli PhD^1^, Floran Sahiti MD^1,2^, Nina Scholz MD^1^, Vladimir Cejka^1^, Judith Albert^1,2^, Georg Ertl MD^1,2^, Christiane E. Angermann MD^1,2^, Gülmisal Güder MD PhD^1,2^, Stefan Frantz MD^1,2^, Peter U Heuschmann MD^1,3,4^, Christoph Maack MD^1,2^ , Stefan Störk MD PhD^1,2^

1 Comprehensive Heart Failure Center, University Hospital Würzburg, Würzburg, Germany

2 Department Medicine I, University Hospital Würzburg, Würzburg, Germany

3 Institute of Clinical Epidemiology and Biometry, University Würzburg, Würzburg, Germany

4 Clinical Trial Center, University Hospital Würzburg, Würzburg, Germany

**Table of content**

**page**

**Supplementary methods 3**

Definition of *apparently healthy* status 3

**Supplementary tables** 3

**Table e1**: Age- and sex specific reference values of systolic ejection time (SET) derived 4
from a population-based cohort of individuals free from heart failure

**Table e2**: Age- and sex specific reference values of heart rate corrected systolic ejection 5
time (SETc) derived from a population-based cohort of individuals free from heart failure

**Table e3:** Baseline characteristics of patients admitted to the hospital with acute heart 6 failure (AHF), with and without serial echocardiograms

**Supplementary figure 8**

**Figure e1**: Correlation between systolic ejection time (SET) and $\sqrt[3]{RR-Interval}$ 8

**Supplementary methods**

Definition of *apparently healthy* status.

An individual was considered *apparently healthy*, if **none** of the following conditions was present:

- Hypertension: blood pressure ≥140/90 mmHg or anti-hypertensive drug
- Smoking: current or ex-smoker
- Obesity : body mass index >30 kg/m2
- Dyslipidemia: low density lipoprotein ≥190 mg/dL or lipid-lowering therapy
- Diabetes mellitus: HbA1c >6.5% or fasting plasma glucose >7mmol/L or 2h plasma glucose > 11.1 mmol/L
- Known cardiovascular disease

**Supplementary tables**

**Table e1**: Age- and sex specific reference values of systolic ejection time (SET) derived from a population-based cohort of individuals free from heart failure (n= 966 *apparently healthy* individuals; 49±11 years, 61% women)

| **SET [ms]** | **Men** | | | | | **Women** | | | | | |
| --- | --- | --- | --- | --- | --- | --- | --- | --- | --- | --- | --- |
| **Age** [years] | 30-39 | 40-49 | 50-59 | 60-69 | 70-79 | 30-39 | 40-49 | 50-59 | 60-69 | 70-79 |  |
| **N** | 66 | 144 | 92 | 60 | 14 | 100 | 234 | 150 | 89 | 17 |  |
| **P2.5** | 266 | 271 | 276 | 281 | 286 | 272 | 277 | 282 | 288 | 293 |  |
| **P10** | 279 | 284 | 289 | 294 | 299 | 285 | 291 | 296 | 301 | 306 |  |
| **P25** | 291 | 296 | 301 | 306 | 311 | 297 | 302 | 307 | 313 | 318 |  |
| **P50** | 304 | 309 | 314 | 319 | 324 | 310 | 315 | 321 | 326 | 331 |  |
| **P75** | 317 | 322 | 327 | 332 | 337 | 323 | 329 | 334 | 339 | 344 |  |
| **P90** | 329 | 334 | 339 | 344 | 349 | 335 | 340 | 346 | 351 | 356 |  |
| **P97.5** | 342 | 347 | 352 | 357 | 362 | 348 | 354 | 359 | 364 | 369 |  |

P= percentile

**Table e2:** Age- and sex specific reference values of heart rate corrected systolic ejection time (SETc) derived from a population-based cohort of individuals free from heart failure (n= 966 *apparently healthy* individuals; 49±11 years, 61% women)

| **SETc [ms]** | **Men** | | | | | **Women** | | | | |
| --- | --- | --- | --- | --- | --- | --- | --- | --- | --- | --- |
| **Age** [years] | 30-39 | 40-49 | 50-59 | 60-69 | 70-79 | 30-39 | 40-49 | 50-59 | 60-69 | 70-79 |
| **N** | 66 | 144 | 92 | 60 | 14 | 100 | 234 | 150 | 89 | 17 |
| **P2.5** | 278 | 283 | 288 | 293 | 297 | 293 | 298 | 303 | 308 | 312 |
| **P10** | 289 | 294 | 299 | 303 | 308 | 304 | 309 | 314 | 318 | 323 |
| **P25** | 299 | 304 | 308 | 313 | 318 | 314 | 319 | 323 | 328 | 333 |
| **P50** | 310 | 314 | 319 | 324 | 328 | 325 | 329 | 334 | 339 | 348 |
| **P75** | 320 | 325 | 330 | 335 | 339 | 335 | 340 | 345 | 350 | 354 |
| **P90** | 330 | 335 | 340 | 344 | 349 | 345 | 350 | 355 | 359 | 364 |
| **P97.5** | 341 | 346 | 350 | 355 | 360 | 356 | 361 | 365 | 370 | 375 |

P= percentile

**Table e3:** Baseline characteristics of patients admitted to the hospital with acute heart failure (AHF), with and without serial echocardiograms

|  | **Total sample  N=623** | **Patients with  serial echocardiograms  N=134** | **P-value for  serial vs. no serial echocardiogram** |
| --- | --- | --- | --- |
| **Female sex** | 247 (40) | 49 (37) | 0.411 |
| **Age [years]** | 74 (11) | 73 (11) | 0.058 |
| **Body mass index [kg/m^2^]** | 29.1 (6.6) | 29.8 (6.6) | 0.109 |
| **De novo heart failure** | 82 (13) | 21 (16) | 0.363 |
| **Coronary disease** | 275 (46) | 57 (44) | 0.537 |
| **Diabetes mellitus** | 264 (43) | 51 (39) | 0.242 |
| **Atrial fibrillation** | 290 (48) | 63 (48) | 0.919 |
| **NYHA functional class*** |  |  | 0.906 |
| **I/II** | 31 (5) | 7 (5) |  |
| **III/IV** | 581 (95) | 126 (95) |  |
| **Pharmacotherapy for HF (at admission)** |  |  |  |
| **Betablocker** | 436 (70) | 93 (69) | 0.868 |
| **ACEi/ARB** | 314 (50) | 70 (52) | 0.631 |
| **Sacubitril/Valsartan** | 15 (2) | 2 (1) | 0.435 |
| **MRA** | 126 (20) | 34 (25) | 0.094 |
| **Diuretics** | 572 (92) | 124 (93) | 0.730 |
| **NT-proBNP [pg/ mL]** | 4644 (2232, 10003) | 4206 (2165, 7919) | 0.497 |
| **eGFR [ml/min/1.73 m^2^]** | 47 (33, 65) | 50 (35, 68) | 0.141 |
| **Glucose [mg/dl]** | 129 (107, 170) | 125 (107, 129) | 0.663 |
| **Total cholesterol [mg/dl]** | 147 (125, 179) | 142 (118, 163) | 0.045 |
| **Serum sodium [mmol/l]** | 139.2 (4.2) | 139.3 (4.3) | 0.745 |
| **Serum potassium [mmol/l]** | 4.5 (0.6) | 4.4 (0.5) | 0.612 |
| **LVEF [%]** | 46.2 (16.9) | 44.5 (18.5) | 0.198 |

Values indicate n (%), mean (SD) or median (Q1; Q3), as appropriate.
NYHA = New York Heart Association functional class, NT-proBNP = N-terminal pro B-type natriuretic peptide, eGFR = estimated glomerular filtration rate, HbA1c = glycosylated hemoglobin c, LVEF = left ventricular ejection fraction.

*In n=4 patients, NYHA classification was not correctly applicable.

**Supplementary figure**

**Figure e1:** Correlation between systolic ejection time (SET) and $\sqrt[3]{RR-Interval}$


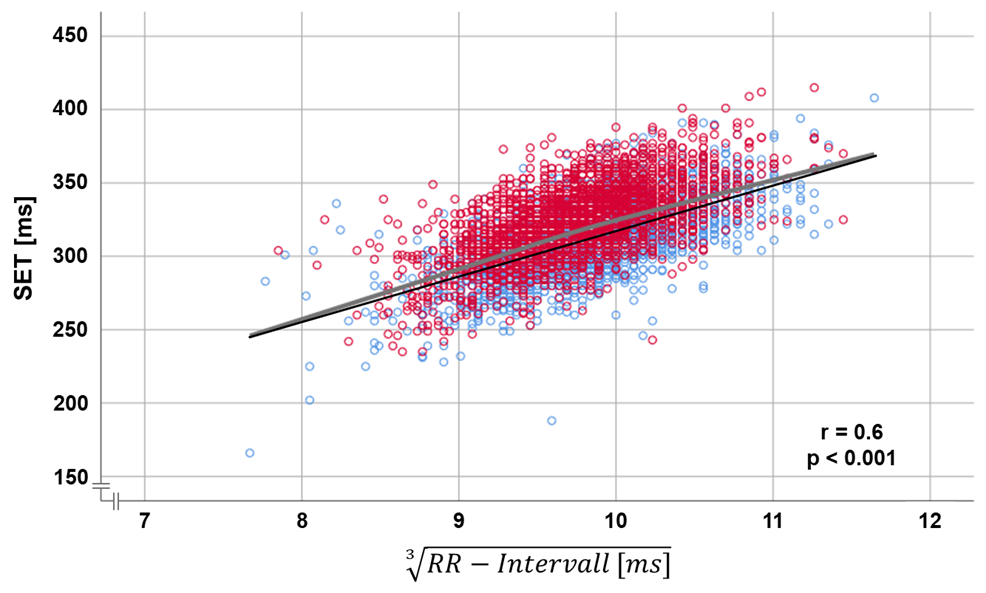

$$\sqrt[3]{RR interval}$$

In both men (blue) and women (red), the best model fit was seen using a Fridericia-like approach, i.e. calculating SETc by $\sqrt[3]{RR interval}$. There, the linear regression line (black) almost converged with the LOWESS regression curve (grey).

RR interval = heart rate/60, SET = systolic ejection time, SETc = heart rate corrected SET, LOWESS = locally weighted scatterplot smoothing.
